# A Proof of Concept Study on Utilising a Non-invasive Microwave Analysis Technique to Characterise Silver Based Materials in Aqueous Solution

**DOI:** 10.1007/s11220-017-0162-y

**Published:** 2017-02-21

**Authors:** Muhammad Ateeq, Andy Shaw, Rob Garrett, Paul Dickson

**Affiliations:** 1 Low Carbon Eco-Innovatory, Faculty of Engineering and Technology, Liverpool John Moores University, Liverpool, L3 3AF UK; 2 Faculty of Engineering and Technology, Liverpool John Moores University, Liverpool, L3 3AF UK; 3 AmesGoldsmith UK Ltd, Acornfield Road, Knowsley Industrial Park, Kirkby, Liverpool, L33 7UF UK

**Keywords:** Microwave sensing, Non-destructive analysis, Novel microwave analysis, Cavity resonator, Silver products, Silver products characterisation, Industrial analysis

## Abstract

This paper reports on the feasibility of using a novel and robust microwave sensing technique to analyse and detect silver materials in an aqueous solution. Two products are tested, namely: silver chloride and silver oxide. The study mainly focused on indicating the difference between them and also any change in the size/size distribution of the sample. A microwave sensor designed previously is utilised to identify the potential of the technique to carry out the analysis. The results are presented as microwave spectrums that are the material response to microwaves. The results have shown that the technique has reasonably indicated the change in material type as well as size distribution. The results also show that these curves are distinguishable and can be related to the material and the change in its size. It is concluded that there is a potential of extending this technique to determine various other properties of silver products. The study suggests a design and development of a bespoke unit as a dedicated analysis tool and to address any anomalies arising from the current feasibility. This will have a huge industrial benefit in terms of cost reduction and time associated with the industrial analysis of silver material.

## Introduction

### Scope of the Study

It is crucial in any manufacturing industry to determine the process variables rapidly and accurately. This proof of concept study focused on introducing a new and innovative method of quality control and assessment of silver materials during the manufacturing process. It investigated the potential of using a non-invasive microwave sensing technique to determine the properties of silver based materials of various types and to instantaneously spot a difference between them. The robustness of the technique demonstrated the potential of it to develop as a real-time option in the situations where timely results are crucial. The investigation was conducted in partnership with AmesGoldsmith UK Ltd, a manufacturer of silver based products. The company was keen on exploring cost effective, robust and reliable ways of characterising the silver based products during the manufacturing process in terms of various properties. The challenging part of the study was to use the microwave sensing technique for the samples in an aqueous solution, which is the case during the manufacturing process of silver products at AmesGoldsmith UK Ltd, based on the fact that microwaves can be absorbed in water due to its high dielectric losses.

Currently, AmesGoldsmith UK Ltd has no method in place to monitor the quality of the product during the manufacturing process. They carry out testing after the manufacturing is complete which is time consuming, laborious to carry out and often the results show inaccuracy and inconsistency in the process. The potential of investigating an efficient technique capable of monitoring the process in real-time would be a significant benefit to the company for quality control and validation of the finished product.

This investigation has not only provided an innovative approach of microwave analysis and its application to characterise the silver based products in aqueous solution but also has introduced a firm ground to the industrial partner with sufficient information on the potential of developing it further to not only monitor the manufacturing process but also to explore its potential to be used to monitor the properties of the material. These properties may include particle size, particle size distribution, contamination, etc. during the manufacturing process. Although this work presents a proof of concept study at this stage, the ultimate aim in the future would be to design and develop a bespoke sensor unit to implement the microwave sensing and monitoring.

## Microwave Sensing Theory, Methodology and Materials

Microwave permittivity measurements and sensing has been much used in the research for material characterisation and detecting the dielectric properties of various substances. The technique has been established over a period of more than 40 years [1, 2]. Due to its accuracy, rapid response, reliability and non-intrusive nature, it has become a rapidly developing technology [1, 3, 4]. It is a low cost measurement technique and has a capability to analyse samples in small or large volumes [5–7]. It is non-ionising in nature, the sensor operates at low power, i.e. at around 0 dBm or 0.001 W (1 mW) and has a good penetration depth in respect of analyte materials [8]. Microwave sensing uses signals between 300 MHz and 300 GHz frequency range. The system can be designed as a portable solution. It is an efficient technique and largely used for the characterisation of materials because it can easily propagate through low-loss substances such as plastics, glass, ceramic, etc. [4, 9, 10]. It is a relatively straight forward technique and the instrumentation for measurements can be setup in minutes with the availability of measurement results in seconds providing real-time data [11]. In addition to the advantages mentioned above, however, the technique has some disadvantages including the requirement of a higher degree of specialisation to interpret the results, simultaneous existence of multiple variables such as temperature, density, moisture, structure, etc. affecting the microwave measurements [12].

Due to its versatile nature, the technique has been implemented in various industrial applications such as to determine the dielectric properties of various substances [2, 13, 14], to determine the particulate blend [15], in the food industry [16–18], glucose concentration and blood glucose monitoring [19, 20], water, oil and gas industry including multiphase flow monitoring [12, 21], characterisation of construction materials [6, 9, 22], water level measurements, material moisture contents, healthcare industry, etc., to name a few [23, 24].

### Microwave Sensing and Resonant Cavity Theory

Microwave sensors can be designed and developed in various sizes, shapes and types depending on the application type. This includes cavity resonators, Flexible antennas/sensors, patch antennas, etc. [6, 13]. Microwaves use the transmission, scattering, reflection and absorption of electromagnetic waves (EMW) to determine the properties of molecules, materials and related species. The microwave frequency band of the electromagnetic wave spectrum is influenced by the rotational energy of molecules and are closely related to their geometric structure. Hence, any change in the geometric structure, type, etc. of the molecules results in the change in microwave spectrum obtained from its interaction with the materials (solids, liquids, gases and suspensions) [9]. The underlying principle of microwave interaction with the material is that the change in the permittivity or dielectric properties leads to the change in the spectrum obtained which in turn is dependent on the molecular structure of it. This means that any change in the molecular structure causes the change in the permittivity of the material [8, 25].

Permittivity is a measurement of change in an electric field due to the presence of the material. It is dependent on the material’s ability to polarise in response to the applied field. The microwave analysis technique can be used to provide unique signal spectrums of two important quantities, i.e. a reflected signal also known as reflection coefficient (S_11_) and a transmitted signal also known as transmission coefficient (S_21_). The Vector Network Analyser (VNA) can be used to generate microwave signals. Since permittivity relates to the material’s ability to transmit an electric field, it is a complex quantity and changes with changing frequency. It takes into consideration both the energy stored by the material called dielectric constant *ε*′, as well as any losses of energy termed as dielectric loss factor *ε*″. These two quantities together are called scattering parameters also referred to as S-parameters. By considering how these parameters change at discrete frequency intervals, the change can be linked to the material type, its composition, concentrations of the constituents, size/size distribution, etc. in the sample [8, 24, 26].

In the current feasibility study, a cavity sensor was utilised as a sensing object. The cavity sensor resonates when the applied electric and magnetic field forms a standing wave pattern inside the cavity resonator. The cavity resonators can have a single mode or multiple modes existing inside at one time. Each of the modes that exist inside the cavity has its own resonant peak/frequency. A quality factor *Q* is used to refer to the quality of the resonant peak, the sharper the peak the more readily the sample can be analysed which in turns improve the accuracy of the sensor. Three fundamental modes in the cylindrical cavity are TM_010_, TE_111_ and TE_011_. The transverse magnetic (TM) mode has its electric component in the direction of the propagation of wave whereas the transverse electric (TE) mode has its magnetic component in the direction of propagation of wave [27]. The resonant frequency in the cylindrical cavity for TM_mnl_ mode can be calculated using the Eq. (1).

1 $$f_{mnl} = \frac{c}{{2\pi \sqrt {\mu_{r} \varepsilon_{r} } }}\left[ {\left( {\frac{{p_{nm} }}{b}} \right)^{2} + \left( {\frac{l\pi }{d}} \right)^{2} } \right]^{1/2}$$

where *ɛ*_*r*_ is the relative permittivity of the material, *μ*_*r*_ is the relative permeability of the material, *c* is the velocity of light, *d* is the depth of the cavity, *b* is the radius of the cavity, *p*_*nm*_ is the nth root of the Bessel function of the mth order.

### Design of the Resonant Cavity and COMSOL Simulations

The feasibility study used an aluminium cylindrical microwave cavity resonant structure to study the properties of various silver materials in the aqueous solution. It was challenging to study if microwaves can penetrate and characterise the material within the aqueous solution. The sensor was a thin pan cake cylindrical cavity and was designed at the Radio Frequency and Microwave (RFM) Group of Liverpool John Moores University (LJMU). Since it was a proof of concept study, the work explored the potential of using microwave sensing as a technique to monitor the properties of silver material during the manufacturing process in aqueous solution. Hence, the focus was on the technique and not the sensor itself to assess if it can be developed potentially as a technique to carry out the analysis.

**Fig. 1:**
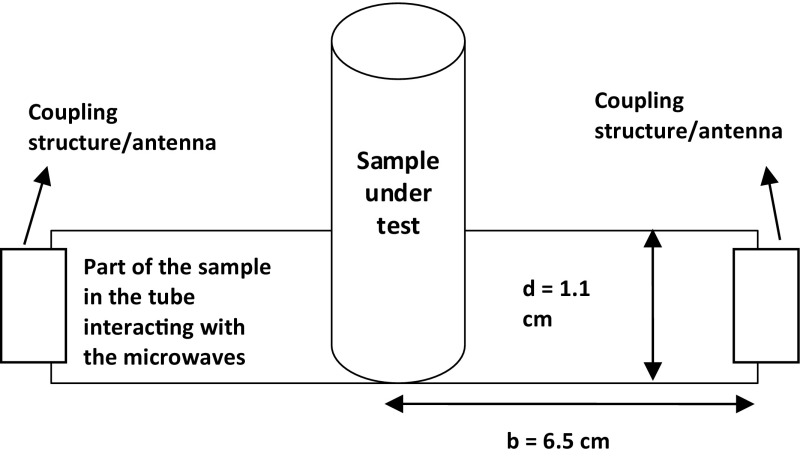
Schematic diagram of the cavity, side view, along with its dimensions

To understand the interaction of microwaves with the sample under test it was important to provide the inner dimensions of the resonator cavity and the arrangement of electromagnetic waves inside the cavity, representing the modes at which the measurements were taken. The modes and the field pattern was simulated using a COMSOL Multiphysics simulation tool and the results are presented. The schematic diagram of the cylindrical cavity’s side view, its inner dimensions, sample and coupling structure is shown schematically in Fig. 1.

**Fig. 2:**
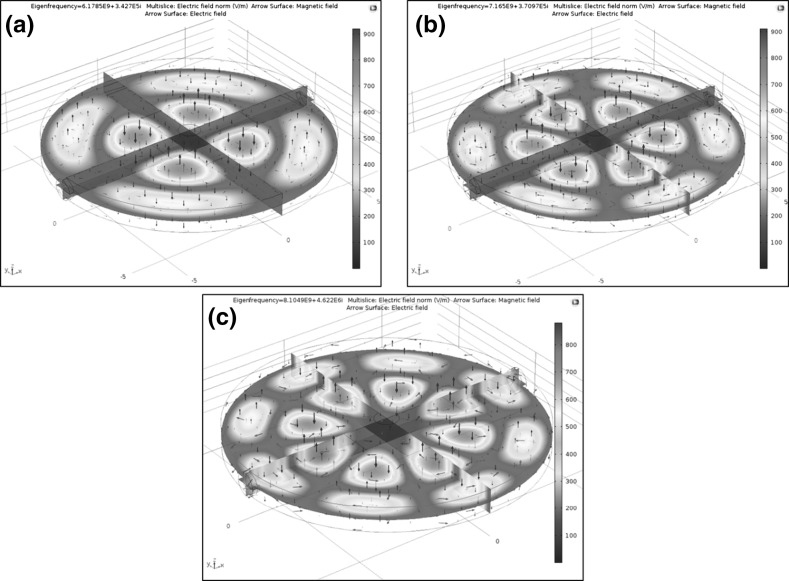
COMSOL simulations of the field pattern and modes generated inside the cylindrical resonant cavity with resonant peaks at **a** 6.1785 GHz, **b** 7.165 GHz, **c** 8.104 GHz

Since, the cavity was not designed for the current work, COMSOL simulations of the resonant cavity filled with air were carried out. The simulations were carried out at the experimental measurement frequencies to compare the experimental and simulated results. These frequencies were identified through a series of experiments conducted and presented in the Sect. 3.2. The frequency for S_21_ measurement was in the range of 6–6.25 and 7.1–7.16 GHz whereas for S_11_ measurement was in the range of 7.95–8 GHz. The experimental resonant peaks in the case of the empty cavity for the measurement frequencies were at 6.1530, 7.1329 and 7.9792 GHz. The simulated resonant peaks were identified at 6.1785, 7.165 and 8.104 GHz. The results of the simulations were used to understand the interaction of microwaves with the test samples at these frequencies to see how the peaks were obtained despite the silver products being in a lossy medium (aqueous solution). The simulations results of the resonant frequencies and their respective modes are presented in Fig. 2.

It can be seen from Fig. 2 that the middle part of the cavity with the sample inlet has a relatively low intensity of electromagnetic field. This low intensity part of the cavity was utilised to carry out the measurements of various samples, i.e. empty cavity, empty tube and silver samples in aqueous solution. From the simulations and experimental results it was found that the modes at which the response peaks were generated at TM_220_, TM_320_ and TM_420_.

### Experimental Setup

**Fig. 3:**
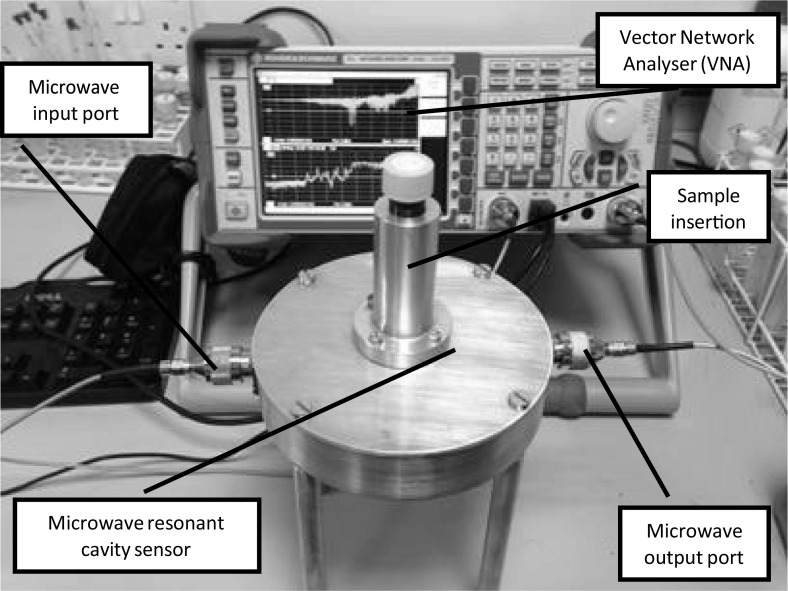
Experimental setup of microwave sensing system, cables, connectors and Vector Network Analyser (VNA)

A cavity resonator can be classed as a black box with EMW (microwaves) entering the cavity from the input port, interacting with the material to characterise it and leaving out of the output ports. The cavity utilised in the current work and the experimental setup of the sensing system is shown in Fig. 3. The cavity was connected to Rodhe & Schwarz ZVL13 Vector Network Analyser (VNA), a microwave source capable of carrying out the measurements between 9 kHz and 13.6 GHz. The power used to carry out the measurements was a minimum, i.e. 0 dBm (1 mW) and the bandwidth of the signal was 10 kHz.

This initial feasibility study made an effort to see how efficiently a microwave sensing technique differentiated between various silver based products inside the aqueous solutions. Further information such as difference in the particle size and a particle size distribution was also indirectly investigated in this study. Although, the size distribution was not quantified at this stage of the feasibility, this area can be explored in detail in the follow up study. The overall size distribution of various samples is presented in Table 1.

**Table 1:** List of the samples analysed along with their description and size range

Sample #	Sample name	Description and sample size (approx.) in (microns)
Samples tested in the microwave sensor		
1	Empty cavity/sensor	N/A
2	Empty sample tube (air)	N/A
3	Silver oxide 1	180–1260
4	Silver oxide 2	180–1260
5	Silver oxide 3	180–1260
6	Silver oxide 4	180–1260
7	Silver chloride 1	240–1500
8	Silver chloride 2	240–1500

### Material and Samples Preparation

**Fig. 4:**
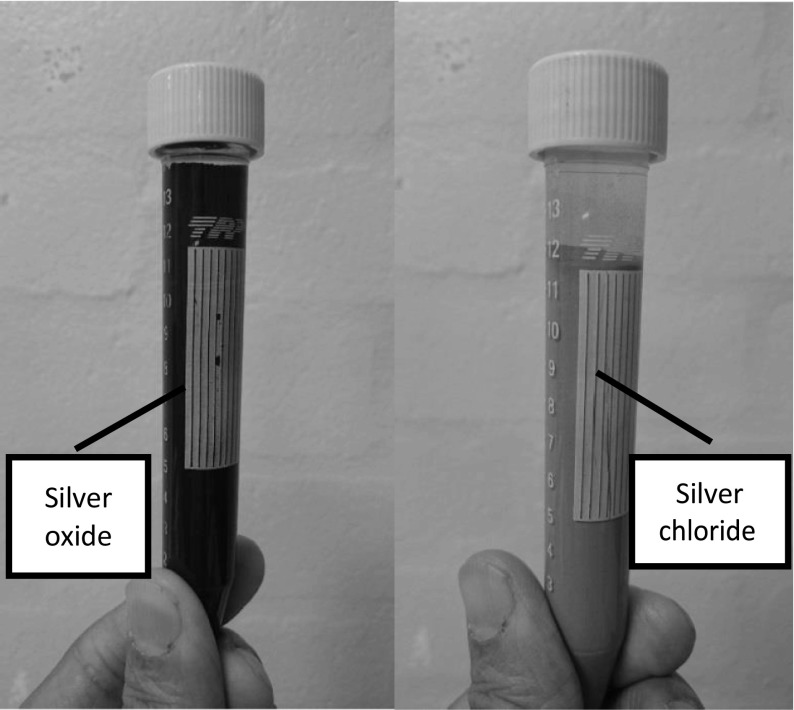
Sample preparation in 15 mL polypropylene tube

**Fig. 5:**
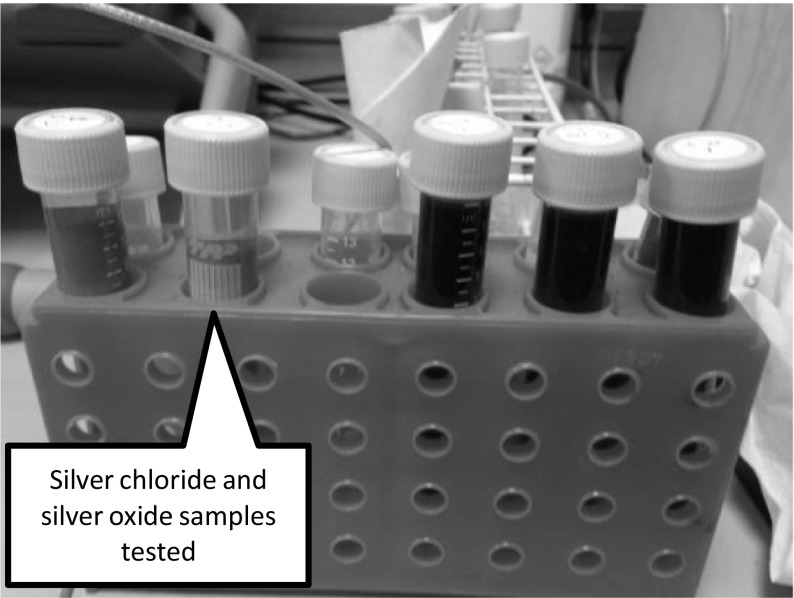
Samples of silver chloride and silver oxide in aqueous solution analysed with microwave analysis

Two types of silver materials, silver oxide and silver chloride, supplied by AmesGoldsmith UK Ltd were used in the study. The samples were prepared and tested in 15 mL polypropylene tubes as shown in Fig. 4. The silver material samples in equal ratio to aqueous solution in the tube was subjected to analysis to ensure the consistency of the measurements. Since the technique is temperature sensitive, the samples were placed at a controlled room temperature of 20 °C for a few hours before testing. The samples are shown in Fig. 5.

The samples tested are listed in Table 1 along with their description, size range and type. It can be seen that a total of 8 samples were analysed. Two control samples of empty cavity and cavity with empty tube inserted, 4 samples of silver oxide and two samples of silver chloride of the same size range but from a different batch were selected. There was a slight possibility of a difference in the percentage of sizes present in a sample. All the samples were analysed individually. To study further the capability of microwave sensors to determine other relevant parameters in the manufacturing process, a future study is recommended, where a dedicated sensor would need developing to target the specific properties of interest.

### Theoretical Resonant Frequency Calculations

Before carrying out the experimental measurements, it was useful to calculate the theoretical resonant frequencies of the empty cavity at the measurement frequencies using the Eq. (1) from Sect. 2.1. This was to establish theoretically the baseline and to later compare the theoretical results with the experimental measurements. Since the modes at which the simulated and experimental resonant peaks generated are known, Eq. (1) can be used to work out the resonant peaks in each case. The results of the theoretical resonant peaks for the modes TM_220_, TM_320_ and TM_420_ is given in Table 2.

**Table 2:** Theoretical resonant peaks calculations for an empty cavity

Modes	Theoretical resonant frequency, f_mnl_ (GHz), $f_{mnl} = \frac{c}{{2\pi \sqrt {\mu_{r} \varepsilon_{r} } }}\left[ {\left( {\frac{{p_{nm} }}{b}} \right)^{2} + \left( {\frac{l\pi }{d}} \right)^{2} } \right]^{1/2}$
TM_220_	6.1829
TM_320_	7.1699
TM_420_	8.1277

## Results and Discussion

### Influence of Silver Oxide on Aqueous Solution

It was important to study the influence of silver in aqueous solution by measuring the dielectric properties of silver product. This was also to help in making comparisons between the experimental and simulated results (presented in Sect. 3.2.3). To give an indication of the accuracy of results, dielectric measurements of only the silver oxide sample was measured. For this purpose, resonant cavity method as described in [27] was used. The dielectric constant and dielectric loss was calculated using Eqs. (2) and (3).

2 $$\varepsilon_{r}^{\prime } = \frac{{V_{c} \left( {f_{c} - f_{s} } \right)}}{{2V_{s} f_{s} }} + 1$$

3 $$\varepsilon_{r}^{{\prime \prime }} = \frac{{V_{c} }}{{4V_{s} }}\left( {\frac{1}{{Q_{s} }} - \frac{1}{{Q_{c} }}} \right)$$

In the above equations, index *c* is for the empty cavity, index *s* is for the cavity loaded with sample and *v* is the volume. The result obtained for the dielectric constant and dielectric loss of the silver oxide sample in aqueous solution is presented in Table 3.

**Table 3:** Dielectric property measurement of silver oxide sample

Frequency of measurement (ghz)	Temperature (°C)	Dielectric constant ($\varepsilon_{r}^{\prime }$)	Dielectric loss ($\varepsilon_{r}^{{\prime \prime }}$)
3.63–3.66	18	1.405	0.0822

Previous study by Ateeq et al. [28] has shown the study of microwave interaction with silver based products in the form of powders such as silver oxide and silver nitrate. The trend in the frequency change for both the S_21_ and S_11_ observed in the currents study, the results of which are presented in Sects. 3.2.1 and 3.2.2, was different to [28] in the case of silver chloride for both the S_11_ and S_21_ measurements. The behavior of silver oxide samples on the other hand was found similar to the [28] in the case of S_11_ measurements. The influence of the aqueous solution itself is minimal, specifically in the case of S_11_ measurements (microwave response) for silver oxide and the microwave frequency change shows the strong characteristic response of silver oxide powder as noticed in [28]. Silver compounds such as silver oxide is insoluble in aqueous solution (water) and extremely stable [29]. This also indicates that why the silver materials analysed in the current investigation have strong influence on the dielectric constant value measured and microwave response with least interference effect from the aqueous solution they are in.

### Experimental Procedure

**Fig. 6:**
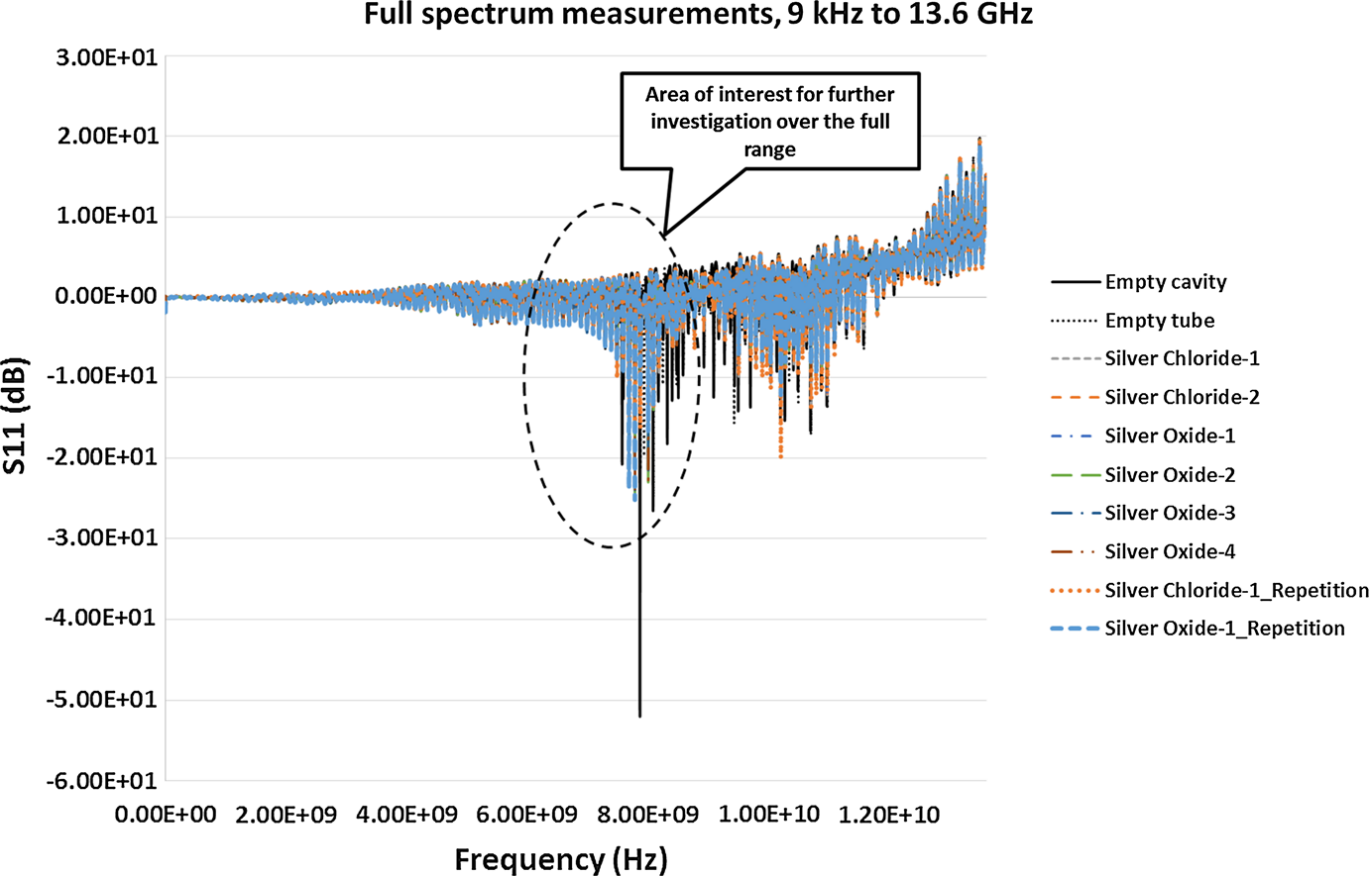
S_11_ measurements (dB), showing the reflection co-efficient measurements between 9 kHz and 13.6 GHz frequency range

Initially the sensor measurements of both S_11_ (reflection co-efficient) and S_21_ (transmission coefficient) was carried out over the full range of spectrum, 9 kHz–13.6 GHz, with the instrument calibrated. This was to identify and highlight the resonant peaks and the area of interest for further investigation and detailed experimental analysis. The frequency was then narrowed down to a small range depending on the response of the material to microwaves. Figure 6 shows the output of S_11_ between 9 kHz and 13.6 GHz frequency.

After careful analysis of the full spectrum it was found that 6–9 GHz section of the spectrum can be further investigated and is encircled in Fig. 6. The selection was based on the more prominent response of microwave interaction with the material around this frequency when looked at carefully. The frequency below 6 GHz didn’t show much of the change when various samples were tested and had higher noise. Frequency above 9 GHz had higher order modes and complexity of analysis associated with it which may require advance analysis techniques to be applied before adequate results are obtained.

**Fig. 7:**
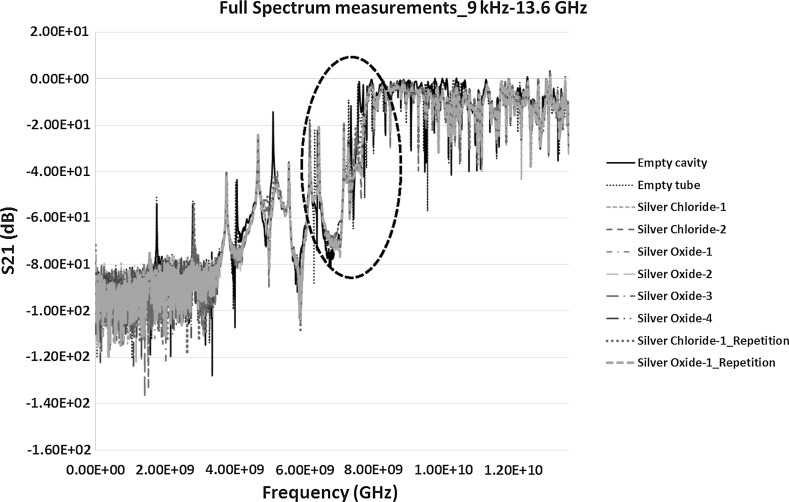
S_21_ measurements (dB), showing the transmission co-efficient measurements between 9 kHz and 13.6 GHz frequency range

Figure 7 shows the S_21_ measurement between 9 kHz and 13.6 GHz. Again, the area of interest was selected based on careful study of the full range frequency response. The encircled section in Fig. 7 shows prominent peaks whereas the frequencies outside the range either exhibited low level of response from various samples or complexities in the measurements. Further results and discussion on the selected sections of the spectrums in Figs. 6 and 7 are presented in the following Sects. 3.2.1 and 3.2.2.

**Fig. 8:**
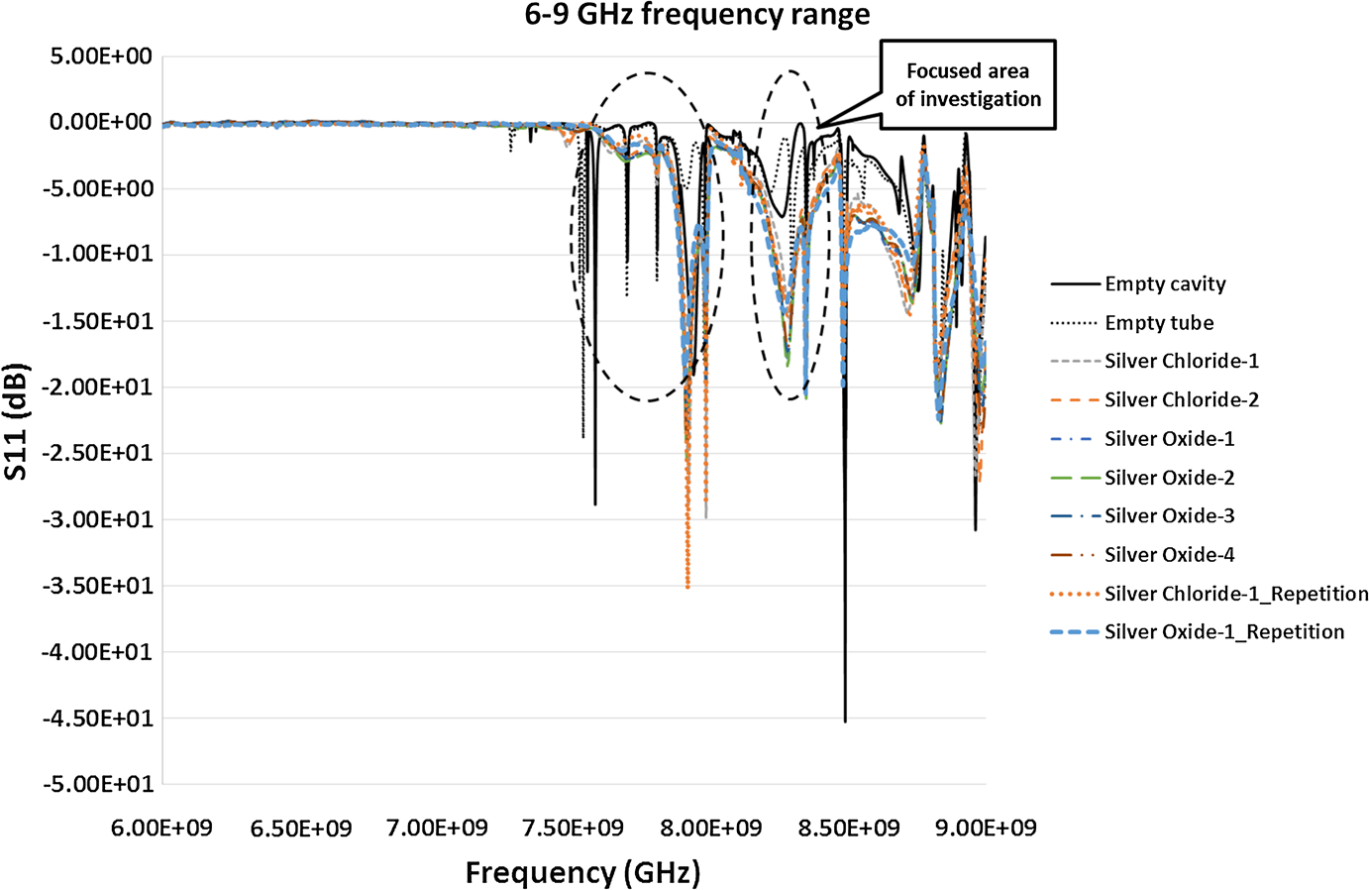
Reflection co-efficient, S_11_ (dB) measurements between 6 and 9 GHz

**Fig. 9:**
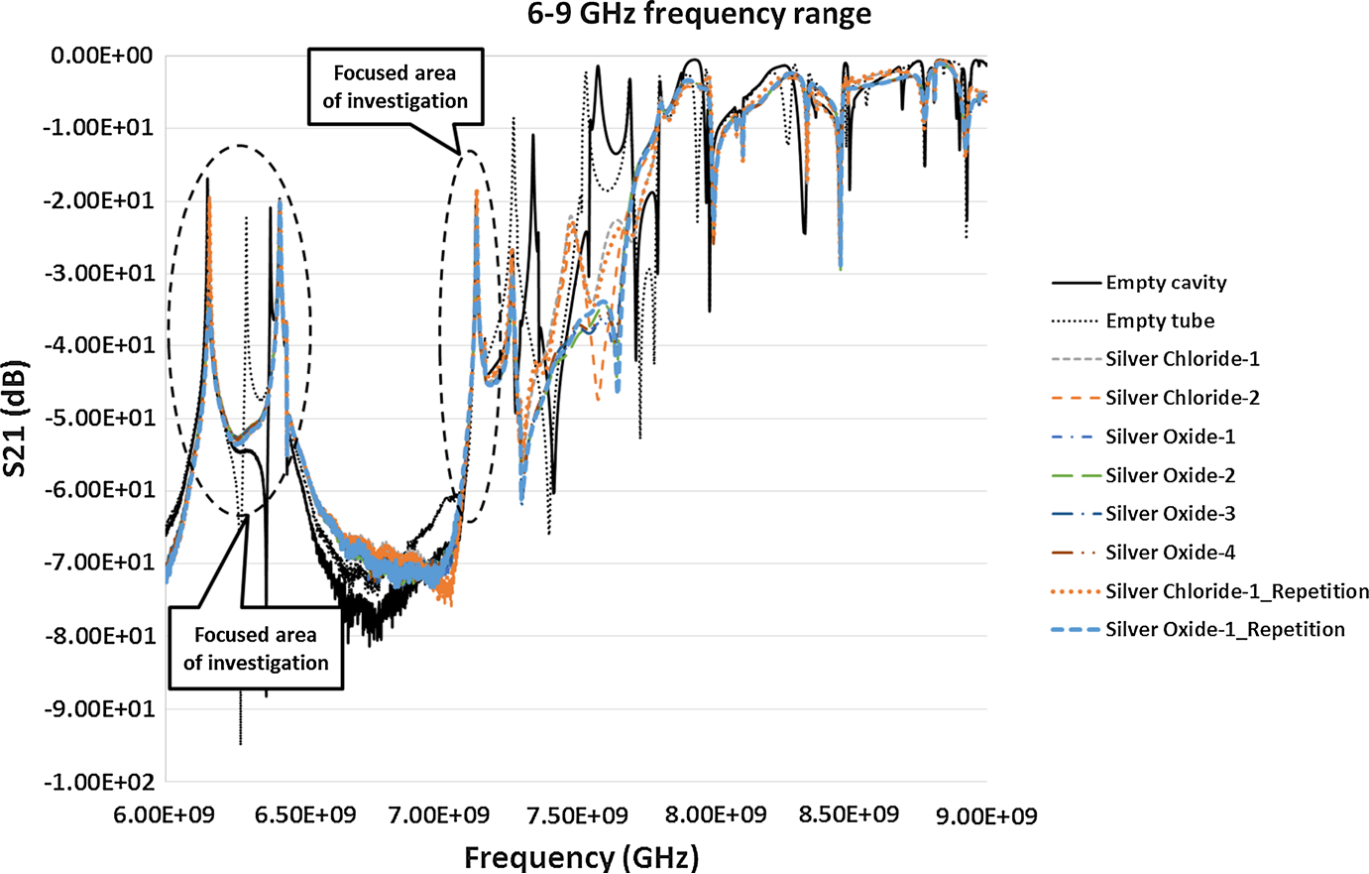
Transmission co-efficient, S_21_ (dB) measurements between 6 and 9 GHz

The VNA instrument was recalibrated between 6 and 9 GHz to take further measurements of the highlighted areas in Figs. 6 and 7. This was to achieve high quality data with higher number of data points to obtain prominent resonant peaks with least error. The results of the S_11_ and S_21_ measurements between 6 and 9 GHz is shown in Figs. 8 and 9 respectively.

**Fig. 10:**
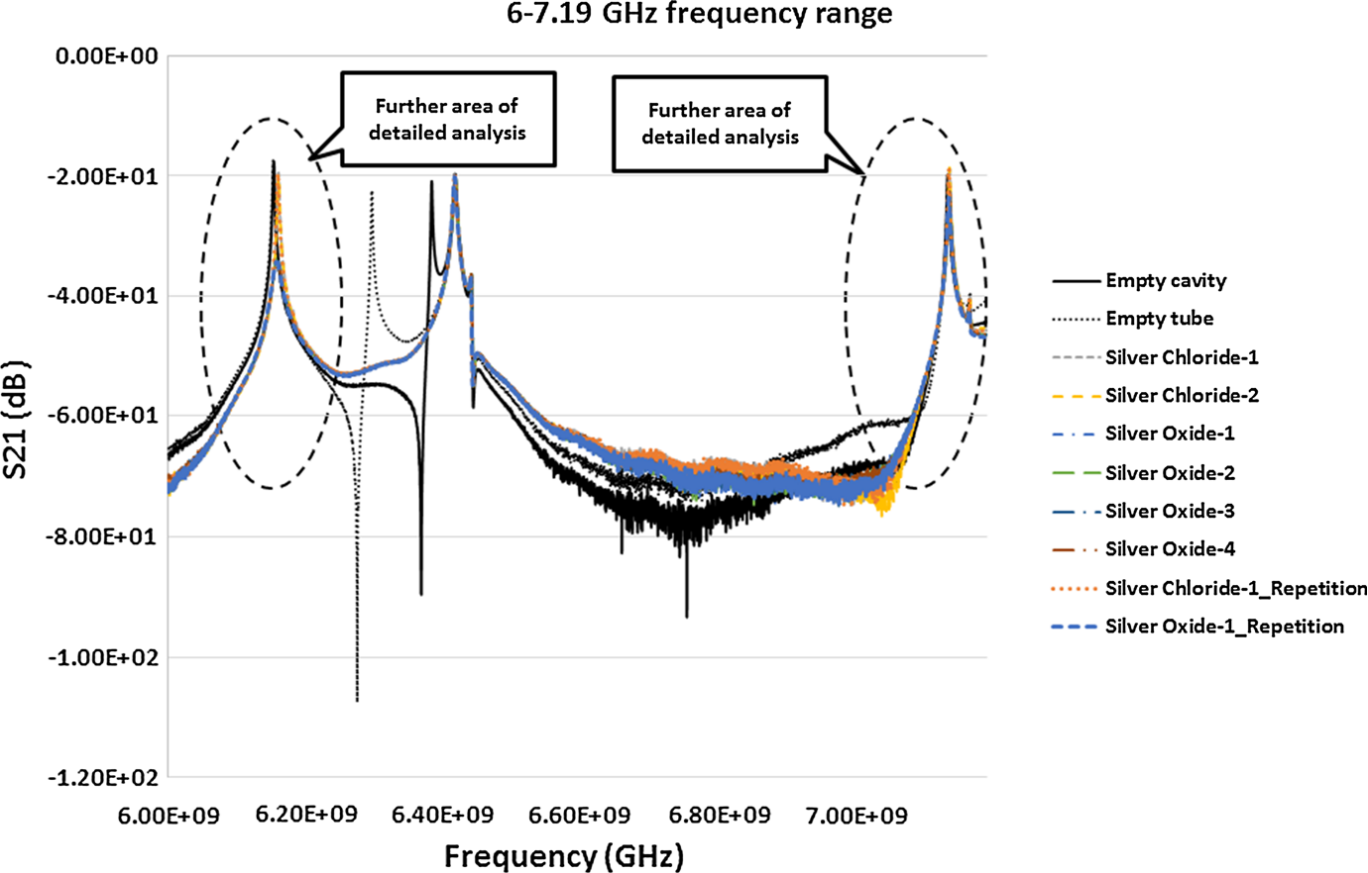
S_21_ (dB) measurements of the material response to the microwaves at the frequency of 6–7.19 GHz

The transmission co-efficient in Fig. 9 was first analysed further because of the prominent peaks and clear response curves. Figure 9 shows the peaks between the frequencies 6 and 7.19 GHz. The range was narrowed down to have a close look at these peaks as in Fig. 10. The results of S_21_ measurements from Fig. 10 highlights two areas of interests each of which was studied separately to investigate the change in the frequency response for each of the sample and to monitor how accurate the response can be for similar samples on repetition. It was also interesting to see how well microwaves can differentiate between samples of silver chloride and silver oxide and how it is compared with the control sample of empty tube in the cavity. The two peaks detected were between 6–6.25 and 7.1–7.16 GHz and are shown in Figs. 12 and 13 respectively and discussed in Sect. 3.2.1.

**Fig. 11:**
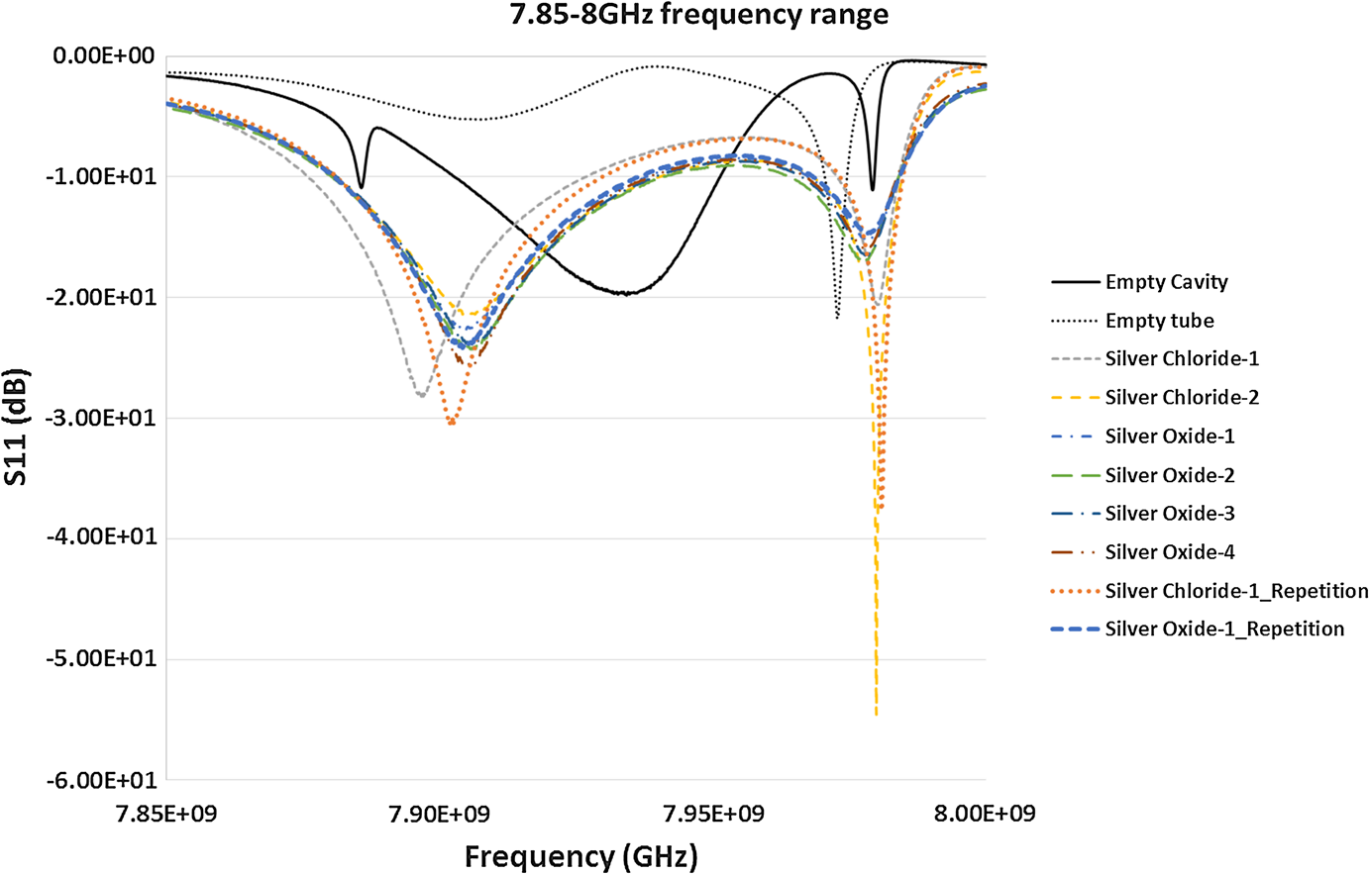
S_11_ (dB) measurements of the material response to the microwaves at the frequency of 7.85–8 GHz

In case of S_11_ measurements in Fig. 8 the results of two resonant peaks detected were more promising between the frequencies 7.85 and 8.5 GHz. However, the peak between 8 and 8.5 GHz range when looked at carefully didn’t give prominent results and was ignored in further analysis. The result between 7.85 and 8 GHz is shown in Fig. 11 and was found worth studying further. Hence, the re-measurements were carried out between this frequency ranges to study the material response to microwaves and is shown in Fig. 14, discussed in Sect. 3.2.2.

#### Micro-analysis of the S_21_ Spectrums in a Narrow Frequency Range

**Fig. 12:**
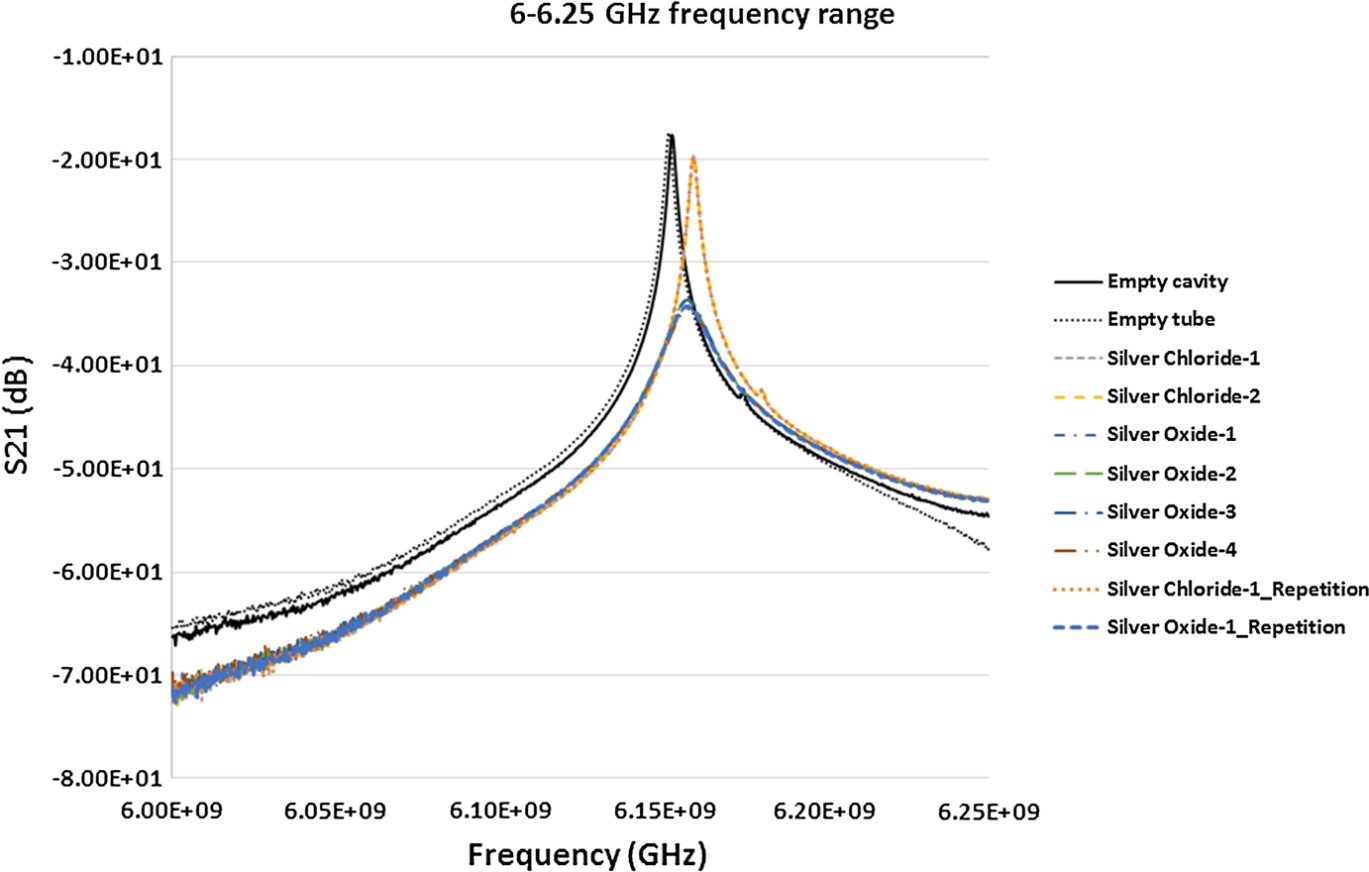
S_21_ (dB) measurements of the material response to the microwaves at the frequency of 6–6.25 GHz

By analysing Fig. 12 it is observed that the peak frequencies (resonant frequency) of the empty cavity and the empty tube in a cavity (taken as a control sample) are at 6.1530 and 6.1521 GHz respectively. The resonant peak of the empty cavity obtained in the experimental measurement at 6.1530 GHz was relatively comparable to the theoretical resonant frequency of 6.1829 GHz presented in Table 2. The comparison of the rest of the samples were then performed with the control sample of empty tube to see the shift in the frequency and change in the amplitude (if any). By analysing the silver chloride-1 and silver chloride-2 samples, the peaks were identified for both at the frequency of 6.1595 GHz. Scientifically, it is very important that the analysis technique should produce repeatable results. Hence, the sample of silver chloride-1 was repeated and the resonant frequency was identified at 6.1598 GHz. It should be noted that when the sample was re-measured for repeatability the side of the tube facing the incident microwave signal may have slight differences in the size distribution of particles compared to the previous measurements. Microwave analysis is sensitive enough to detect this kind of change in the distribution.

When the measurements for the silver oxide samples was analysed, the peaks for the samples silver oxide-1, silver oxide-2, silver oxide-3 and silver oxide-4 were detected at the frequencies 6.1580, 6.1574, 6.1580 and 6.1583 GHz respectively. On repetition of the sample silver oxide-1 the peak frequency was at 6.1577 GHz. This slight change again could be attributed to the change in the size distribution facing the incident wave and may need further investigation to prove it. The results of the above measurements and Fig. 12 show that:

- The shift of the frequency from the empty cavity to the empty tube sample was 0.9 MHz to the left showing the introduction of polypropylene tube in the cavity. The rest of the comparison was performed between the control empty tube sample and the silver material samples.
- The shift for both the silver chloride samples was around 7.4 MHz to the right showing an increase in the frequency. It is significant and shows the demonstrable shift in the frequency and distinction between the empty tube and silver chloride sample in aqueous solution. When looking at the result of repeated sample, the shift from the control sample was around 7.7 MHz. Also the difference between the original sample and re-measurement was minute, i.e. 0.3 MHz which is pretty negligible and could be built into the design of a bespoke unit.
- The shift for silver oxide samples was in between 5.3 and 6.2 MHz to the right from the control sample, i.e. empty tube. This difference is again significant, and the technique being sensitive, is large enough to demonstrate the potential of the technique to analyse silver oxide samples in aqueous solution. When this shift was compared with silver chloride sample the difference was more than 2.1–1.2 MHz between them. The peaks for silver oxide samples were between the control and silver chloride samples. Again this value is large enough in comparison to the sensitivity of the technique to demonstrate the potential of differentiating between the two different types of Silver products, in this case silver chloride and silver oxide.
- In addition to the change in the frequency, significant change in the amplitude was observed between the two silver types as well as in comparison of them to the control sample of empty tube as seen in Fig. 12.

**Fig. 13:**
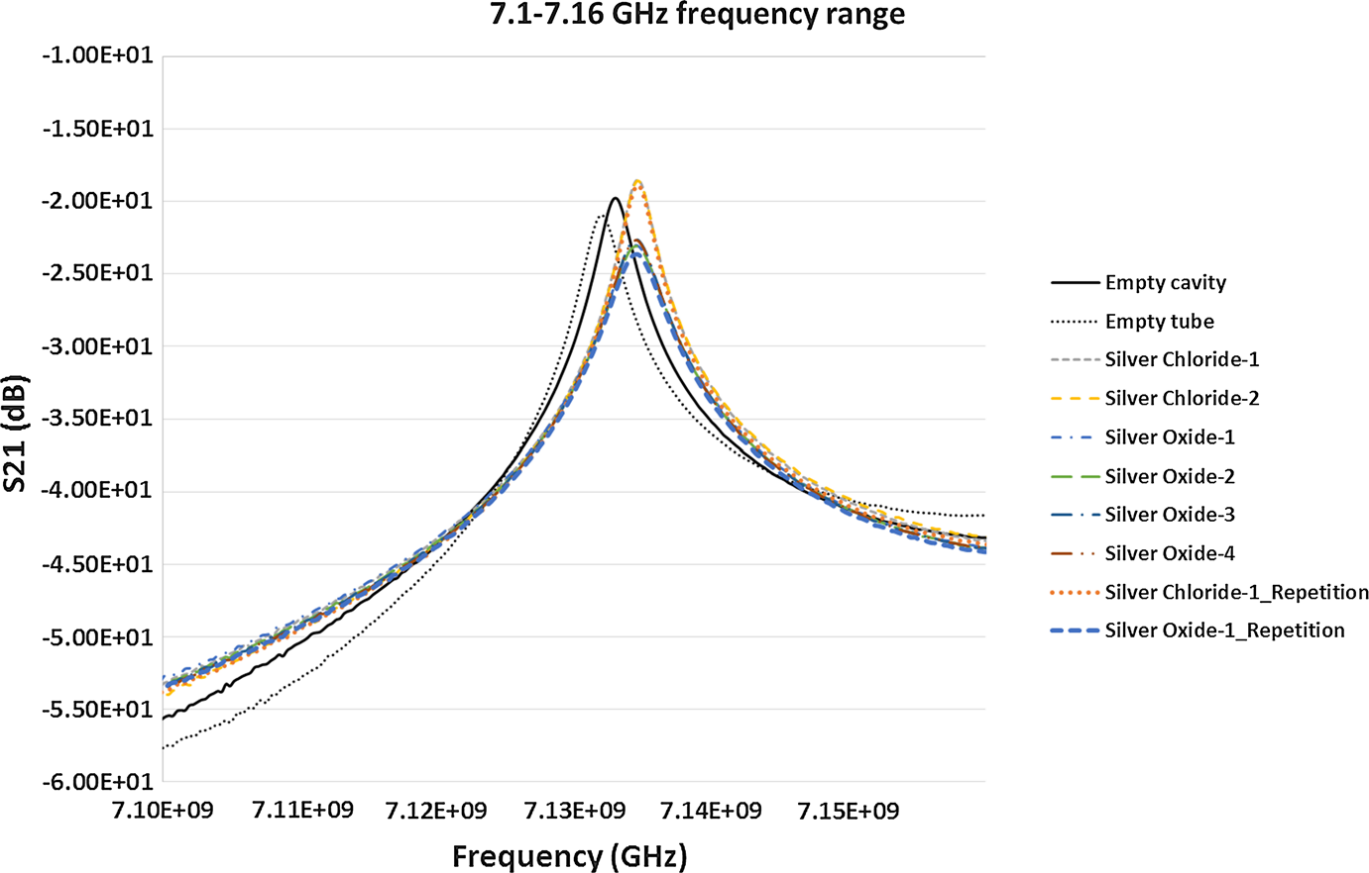
S_21_ (dB) measurements of the material response to the microwaves at the frequency of 7.1–7.16 GHz

When the S_21_ measurements between 7.1 and 7.16 GHz in Fig. 13 were analysed it can be observed that the empty cavity and empty tube samples have the resonant peaks at 7.1329 and 7.1320 GHz respectively. In comparison, the theoretical resonant peak of the empty cavity was calculated at 7.1699 GHz. When the analysis on the shift of silver chloride samples was carried out the peaks for both the silver chloride-1 and silver chloride-2 samples as well as the repeated silver chloride-1 sample were detected at the frequency of 7.1347 GHz. For all the four silver oxide samples including the repeated silver oxide-1 sample, the resonant peaks were detected at the frequency of 7.1343 GHz. From the results obtained it can be stated that:

- The shift in the frequency of silver chloride samples in comparison to the control sample is around 2.7 MHz towards right showing the increase in the frequency. Again, the shift is significant and along with the change in amplitude in comparison to the control sample demonstrates that the microwave sensing technique is detecting the difference between the silver chloride and control sample.
- The shift in the spectrum of silver oxide to the right was around 2.3 MHz which is a high reading on the basis that the technique is very sensitive. The difference between the silver chloride and silver oxide sample is around 0.4 MHz. Although it is tiny and could be negligible, in conjunction with the amplitude change in comparison to silver chloride sample could prove significantly important to differentiate between the two types of silver products. This capability of considering both the frequency shift and amplitude needs to be built into the design of the sensor.
- As stated earlier, in addition to the change in the frequency observed, a significant difference in the amplitudes between the samples exists. The bespoke sensor for the purpose can be designed to take this into consideration within an acceptable range of amplitude change.

In summary, the results between the frequency ranges of 6–6.25 GHz were more effective in comparison to the frequency range of 7.1–7.16 GHz in differentiating between the silver products. In addition, the results in Figs. 12 and 13 also shows that the two parameters, i.e. frequency and amplitude, can be used together in the sensor detection capability to differentiate between various types of silver samples. The results show another important parameter called the quality factor ‘*Q*’ of the cavity. Figure 12 shows a high quality factor of the cavity in the given frequency range in comparison to slightly lower quality factor in the case of Fig. 13. It can be observed that high *Q* factor cavity can increase the sensitivity of detection as well as characterisation of the material and can easily be analysed.

The initial results discussed above show the potential of the microwave sensing to be developed as a technique to analyse Silver products in aqueous solution. However, a bespoke sensor unit is required to minimise the error and to maximise the detection capability by keeping the sensitivity at balance. This can be achieved through step by step modelling and the design of a microwave sensor.

#### Micro-analysis of the S_11_ Spectrums in a Narrow Frequency Range

**Fig. 14:**
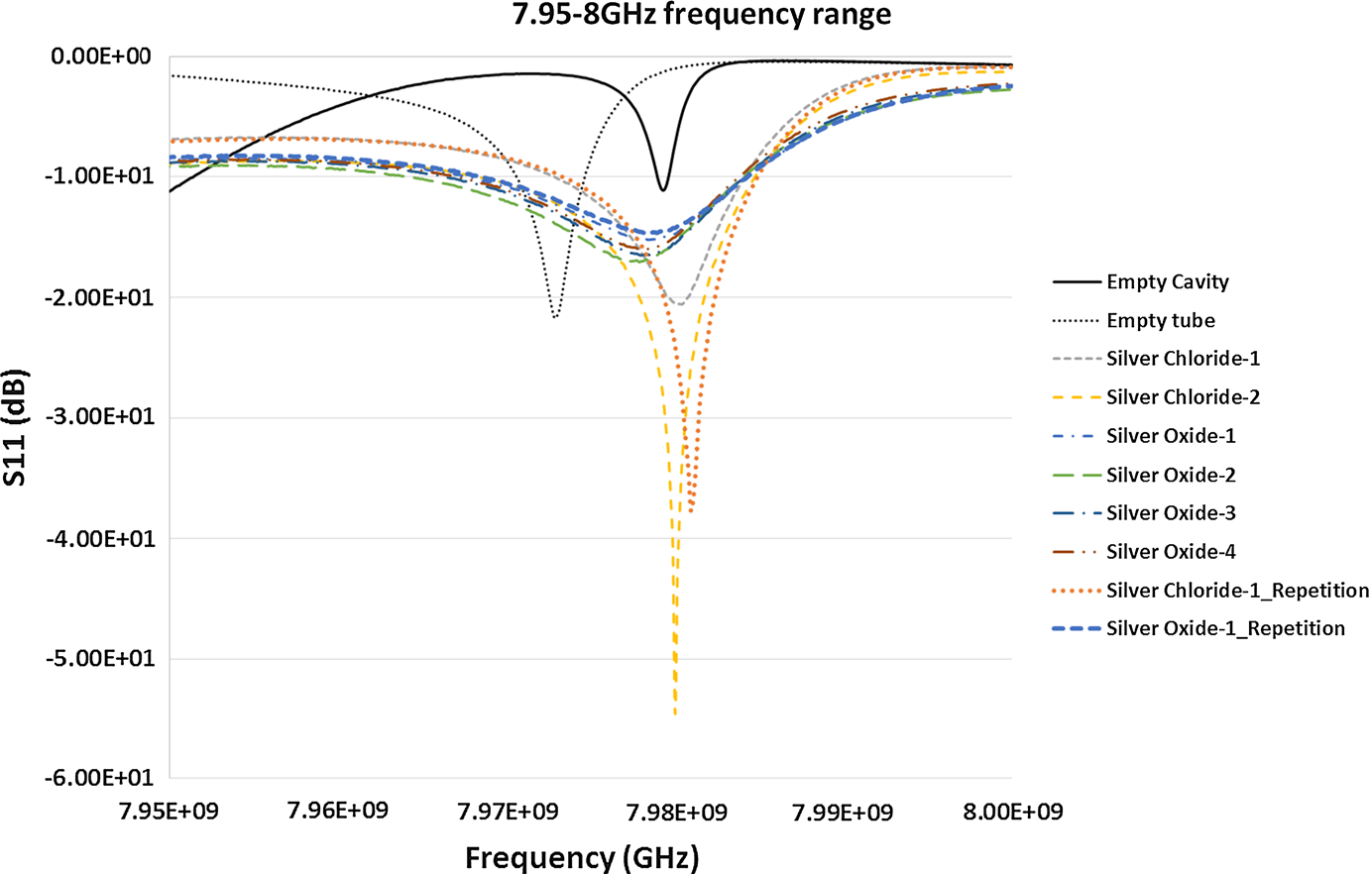
S_11_ (dB) measurements of the material response to the microwaves at the frequency of 7.95–8 GHz

Although prominent but less consistent results in terms of repetition were obtained from S_11_ measurements, it was worth considering the effectiveness of microwave sensors using the reflection co-efficient measurements. The re-calibrated measurement results for the frequency 7.95–8 GHz is shown in Fig. 14. After careful analysis of Fig. 14, it can be seen that the response of each sample to microwaves is slightly different. The shifts in the peaks are consistent with the S_21_ measurements, however, there is a change in the amplitude with low quality factor ‘*Q*’ measurement results, specifically in the case of silver oxide.

As seen in Fig. 14, the resonant frequency for the empty cavity and control sample of empty polypropylene tube was at 7.9792 and 7.9728 GHz respectively. It shows a shift of around 6.4 MHz to the left for the empty tube sample in comparison to the empty cavity. The measurements of silver chloride-1 and silver chloride-2 exhibited a peak frequency of 7.9801 and 7.9800 GHz with a minute difference of 0.1 MHz. This is approximately a shift of 7.3 MHz to the right in comparison to the controls ample which is again significant. When the measurement of silver chloride-1 was repeated the peak frequency was detected at 7.9810 GHz. It is a shift of 1 MHz from the first measurements of silver chloride-1. This shows that the repeatability in the case of S_11_ measurements need to be further examined and careful consideration should be given to the design of the new sensor. However, it would be of concern if the measurements need to be carried out using S_11_ (reflection co-efficient) mode which may not be the case for this application because repeatable and consistent results are obtained in the case of S_21_ measurements. Hence, better results might be achieved if the design of the sensor is carried out around the transmission coefficient measurements. For the silver oxide, the measurements results of silver oxide-1, silver oxide-2 shows peaks at 7.9784 GHz whereas silver oxide-3 and silver oxide-4 shows peaks at the frequencies 7.9785 GHz. When the silver oxide-1 sample was repeated the peak value is at 7.9784 GHz which is similar to the original. The shift in the case of silver oxide from the control sample is around 5.7 MHz which shows the capability of the sensor detection and is very reasonable in terms of resonant peaks occurrences.

In case of S_11_ measurements, the only difference observed was the change in the amplitude of every sample. If it is important to design a sensor around S_11_ detection the sensitivity and accuracy can be looked into and could possibly be reduced by the optimum design of the bespoke unit.

The theoretical resonant peak for an empty cavity calculated in Table 2 was at 8.1277 GHz. This varied in comparison to the experimental value. However, as mentioned above the S_11_ measurement may not be accurate and may not be a good choice for the future sensor development for characterising the silver products in aqueous solution.

#### Comparison of Simulated and Experimental S_21_ Results

**Fig. 15:**
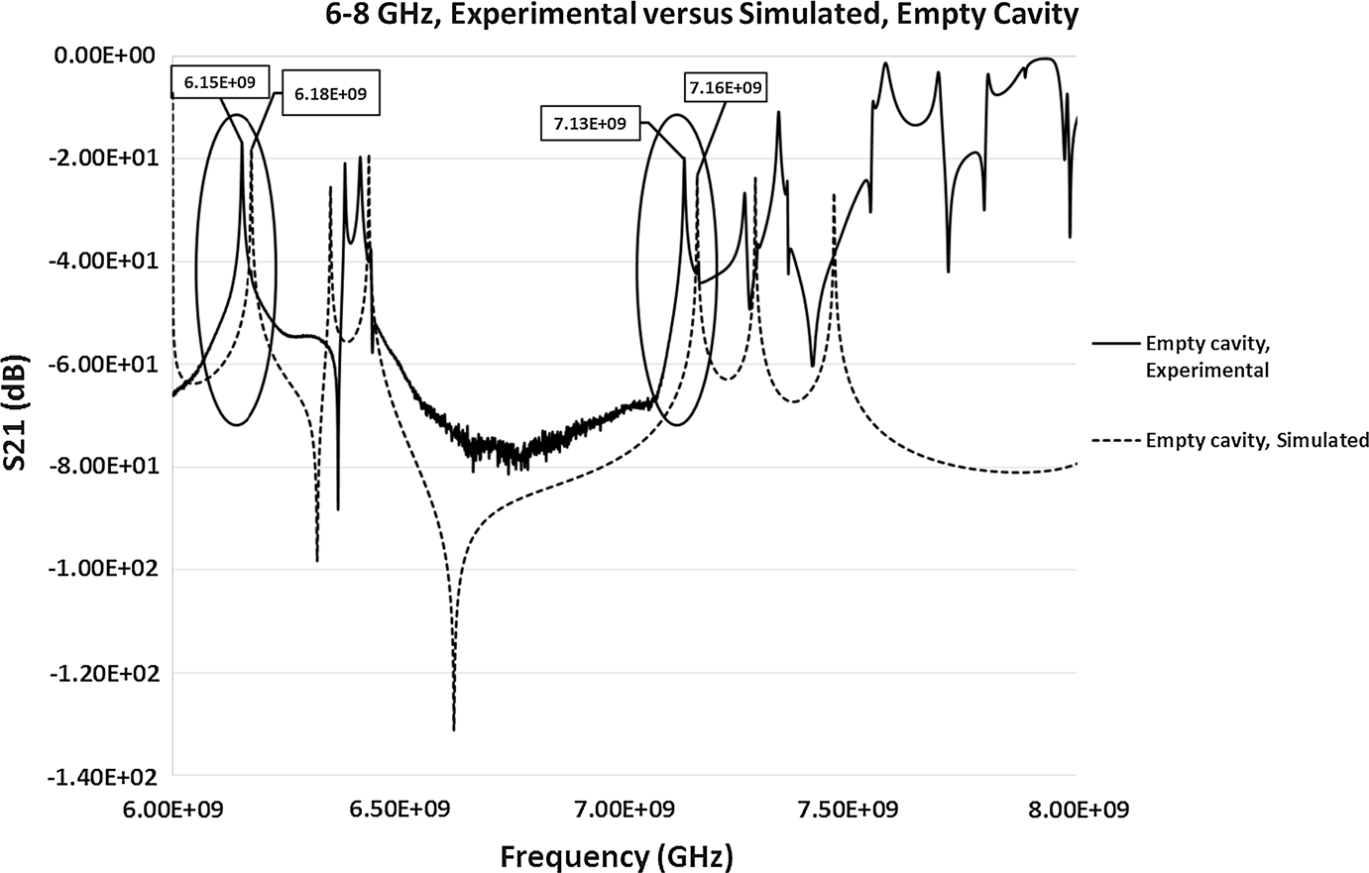
Simulated and experimental S_21_ measurements of the empty pan cake cavity sensor between the frequencies of 6 and 8 GHz

**Fig. 16:**
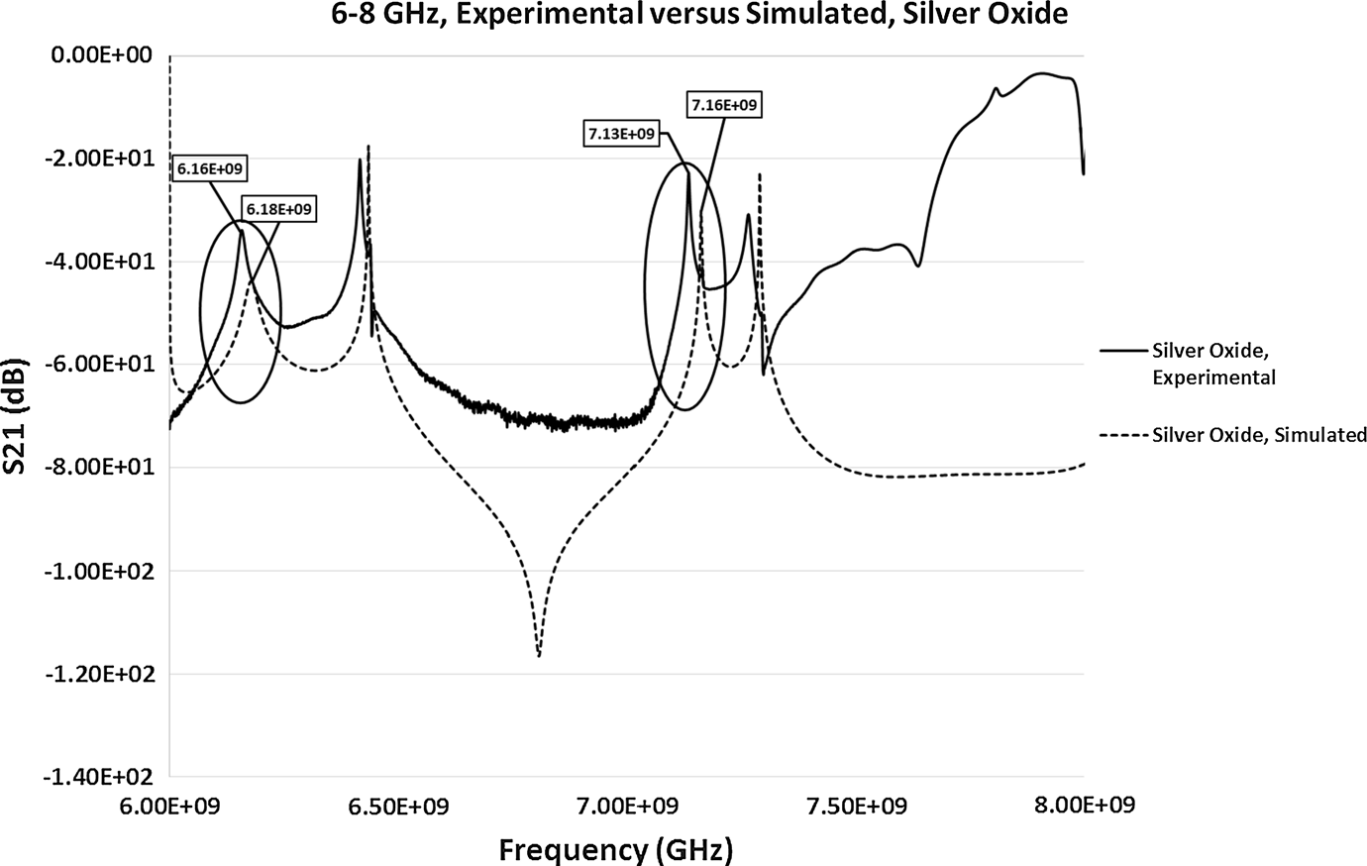
Simulated and experimental S_21_ measurements of the cavity loaded with the silver oxide sample between the frequencies of 6 and 8 GHz

To make a direct comparison between the experimental and simulated results to evaluate how close they are, full Electromagnetic simulations on the S-parameters were carried out in between the frequency range of 6–8 GHz for the simulated S_21_ parameter. Only the S_21_ parameter was considered due to the evident difference in the experimental results of the response frequencies to various silver materials. Simulated S_11_ parameter was measured but the resonant frequency was detected at around 8.1 GHz in comparison to the experimental resonant frequency of 7.9792 GHz. This was a significant difference between the experimental and simulated calculations, similar to the variation between the theoretical and experimental calculations. Hence, to avoid the confusion to the reader the results are not here. For the purpose of this study only two samples were simulated for S_21_, i.e. empty cavity, and cavity loaded with the silver oxide sample. The measured value of the permittivity (discussed in Sect. 3.1) of silver oxide sample was used in the simulations. The results of both the simulated and experimental S_21_ parameters for the empty cavity and loaded cavity with silver oxide sample are presented in Figs. 15 and 16 respectively. This is to make the direct comparison possible and to identify the differences between the simulated and experimental results. Figure 15 shows the comparison of the simulated with the experimental resonant frequencies (highlighted in Fig. 15) of an empty cavity. The simulated resonant frequencies were identified at 6.18 and 7.16 GHz approximately, which closely corresponds to the experimental results of the resonant peaks at 6.1530 and 7.1329 GHz for an empty cavity. The additional peaks in the simulations, differences in the experimental and simulated signal at higher frequencies and the slight difference in the amplitude of the simulated signal in comparison to the experimental results could be due to the way coupling devices has been modelled. Similarly, for the cavity loaded with silver oxide sample, the simulated resonant peaks (highlighted in Fig. 16) were identified at approximately 6.18 and 7.16 GHz, which is close to the experimental results of 6.1580 and 7.1343 GHz. The simulated results could possibly be improved by giving design considerations to the modeling of coupling devices.

#### Repeatability of Samples

Repeatability of both the silver oxide and silver chloride samples was tested as seen in Figs. 6, 7, 8, 9, 10, 11, 12, 13 and 14 by measuring the silver oxide sample five times and silver chloride sample twice. The results were reasonable with a consistent frequency response of microwave to the silver material. Since the study is in feasibility stage and indicates some good repeatability of the technique, detailed repeatability of the microwave response to silver materials will be tested in the following study along with additional statistical analysis. The plan is to design and develop a bespoke sensor that will look into additional analysis such as detailed repeatability, measurement of silver materials and their concentration in the aqueous solution as well as study on various aspects of resonant frequency such as dielectric constant and loss factors.

### Quality Factor (Q value) Calculations

The quality factor estimates the quality of the results obtained. It provides an indication of the accuracy of the results. High Q value will have a high accuracy and narrow bandwidth of the cavity resonator. The Q value can be calculated from the transmission and reflection coefficient measurement curves, i.e. S_21_ and S_11_ parameters and is represented by Eq. (4).

4 $$Q = \frac{{f_{0} }}{{\Delta f}}$$

where *f*_0_ is the resonant frequency of the signal captured, Δ*f* is the bandwidth of the signal obtained using the cut-off frequencies *f*_2_ − *f*_1_ each of which is obtained by finding a 3 dB amplitude change on either side of *f*_0_. The Q values of the results from Figs. 12, 13 and 14 for S_21_ and S_11_ are presented in Table 4. The results of the Q value for the S_21_ and S_11_ signals for various frequency range show that the S_21_ measurements made at the frequency range of 7.10–7.16 GHz yields the highest accuracy with a best quality output when the cavity is loaded with silver chloride and silver oxide samples. However, from the analysis in Sect. 3.2.1, it is observed that the frequency shifts were more prominent and distinguishable between the control sample (empty tube), silver chloride and silver oxide samples. This concludes that Q value of the cavity should not be the only parameter when designing the cavity sensor. Other factors such as the change in the frequency and amplitude also play a vital role in the design of the sensor and the analysis of the results. In terms of the measurements presented, the results are an indication of this consideration. When designing a bespoke sensor, results from these measurable parameters must be considered.

**Table 4:** Quality factor (Q values) of the measured curves of samples obtained from both the S_21_ and S_11_ signals

Sample tested	Measured signal, S_21_ or S_11_ (dB)	Frequency of measurement (GHz)	Q value
Empty cavity	S_21_	6.00–6.25	3619.41
Empty tube			3618.88
Silver chloride			2587.38
Silver oxide			530.56
Empty cavity	S_21_	7.10–7.16	3424.29
Empty tube			2663.00
Silver chloride			3996.00
Silver oxide			1844.23
Empty cavity	S_11_	7.95–8.00	6689.47
Empty tube			6684.14
Silver chloride			3345.12
Silver oxide			836.10

### Dielectric Constant Results and Its Relationship with the Frequency shift

As discussed in Sect. 3.1, to give an indication of the behavior of silver products in aqueous solution, the dielectric constant value of silver oxide in aqueous solution was measured and was recorded to be $\varepsilon_{r}^{\prime }$ = 1.4051. This value was higher than the dielectric constant value of air inside the cavity, i.e. $\varepsilon_{r}^{\prime }$ = 1 representing that the shift of the frequency supposedly should be to the left of the measured signal of the empty cavity. This seems to be true for the S_11_ measurements (Fig. 14) where the shift is to the left of the empty cavity signal specifically for the silver oxide samples. However, the S_21_ measurements (Figs. 12, 13) doesn’t satisfy this conventional definition of the frequency shift where the shift is usually to the left with increase in the dielectric value of the material. Thus, the relationship between the frequency shift and the dielectric constant value of the samples is not consistent.

This behaviour has been observed by some previous research studies such as Kumar and Sharma [30] and Min et al. [31]. In the case of most materials, a decrease in the dielectric constant value is expected with an increase in the frequency due to the dielectric relaxation phenomena (typical times ~10^−11^ s). This means that at higher frequencies the speed of dipole rotation is insufficient and cannot match with the changing AC bias [31]. However, it may be speculated that for certain materials it is possible that the friction against the dipole motion drops with frequency. If it is assumed that the friction of the dipole is due to the molecular rearrangements of the environment and the bond between them, the timescale of the molecular rearrangements would be slower than the dipole motion. Thus, the friction would become less at higher frequencies causing an inverse effect in the frequency with the change in permittivity. Min et al. [31] also found that most of the materials experience change in the dielectric constant value due to the temperature enhanced molecular mobility and dipole rotation phenomena. For some materials, however, the temperature dependence is significant. In such cases the dipoles are restricted at room temperature and do not follow the AC field closely. When the temperature is increased the molecular motions become easier which causes the reinforcement of the response to the field variation. This results in both the dielectric constant and loss factor to increase. When the temperature is reduced, both the mobility and rotation would be restricted again. During the course of reduction in the temperature, the process may not be simply reversible and could cause some of the molecules and dipoles not to go back to the previous condition and may acquire new equilibrium. Thus, the dielectric constant will increase with rising temperature and will show a decrease at low temperature [31]. In the case of more complex mixes where more than one material is present there might be an accumulation of charges at the interface between the two materials which could result in an interfacial polarisation and subsequently field distortion (Maxwell–Wagner effect). The changes thus become smaller and may cause the system to behave opposite, i.e. increase in the dielectric constant with the frequency at room temperature [32, 33]. In the case of samples measured in this study, an experimental study is required to identify which of the above factors is the main contributor to this particular phenomenon and could make an interesting study to follow. In summary, the material tested could be either frequency or temperature dependent or it could be influenced by both.

## Conclusions and Recommendations

An initial feasibility study was carried out on the use of microwave sensing technique as a potential method to analyse silver materials and their properties in aqueous solution. The scope of this preliminary study was mainly focused on differentiating between various silver products such as silver chloride and silver oxide as well as indirect detection of change in the size distribution between the two products. Microwave sensing was found to be a robust, instantaneous, cost effective and repeatable technique and has a potential to analyse the silver material which is of great benefit to the industry. The results were promising exhibiting accuracy and repeatability (for the theoretical, simulated and experimental values), the attributes required for any industrial application, especially in the case of transmission co-efficient, S_21_ measurements. The results have shown that the technique is capable of detecting various silver based products and display microwave spectrums of them. However, detailed design work is required to develop a dedicated unit with a full potential to not only minimise the error but also to include possibly a detection of multiple properties of the material and linking them back to the material itself. Potentially, the technique can be further developed as an alternative to conventional time consuming physical and chemical testing methods. The biggest advantage would be the cost and time savings associated with the technique if available to the Silver products industry.

A thorough study of smaller sections of microwave spectrum and individual peaks helped in exploring and assessing the potential of developing microwave sensing as a technique to differentiate between the silver samples. This brief investigation also pointed out the capability of extending this technique and carry out further research work to see if the technique can enable the characterisation of the material including particle size, particle size distribution, contamination, etc. to name a few. Some of the conclusions and recommendations are listed below:

- Microwave sensing technique demonstrated its capability to spot individual samples of silver chloride and silver oxide and showed the difference between them. If further research work is carried out, the spectrums captured from the dedicated sensor designed can be stored in a database to create a wealth of microwave spectrums representing certain material properties.
- The differences were not only spotted in the frequency shift but also in the amplitudes between silver chloride and silver oxide samples. It was also found that on repeat measurements of the similar material, i.e. silver oxide-1, silver oxide-2, silver oxide-3, silver oxide-4 or silver chloride-1, silver chloride-2, the amplitude slightly changed, especially in the case of S_11_ measurements. This could be attributed to the variation in the size distribution percentage in the sample or the way the sample(s) was re-inserted, i.e. different side of the tube facing the incident microwave. To develop an effective microwave sensor, both of these parameters should be considered and combined together to relate it to a certain type of sample. Further work in the analysis could also possibly make this technique more intelligent by relating the changes in the microwave response to the specific properties of silver materials. This could be achieved through artificial intelligence techniques.
- At AmesGoldsmith UK Ltd, the silver products produced have fewer size distribution range. However, if a sensor is developed for wider range of particle size and size distribution detection addressing the wider silver based industry, different combinations of particle size and particle size distribution may provide similar resonant frequency and quality factor Q. This can be addressed by implementing advance post analysis/processing techniques such as artificial intelligence and neural networks analysis in the processing of the results obtained to distinctively identify various products and relate them to the products specific properties.
- The simulated and experimental results almost provided comparable resonant peaks at the measurement frequency range, showing the resemblance between the simulated and experimental results. The exception was the measurement of S_11_ parameter that has a simulated resonant peak detected at higher frequency than the experimental result and hence was not considered for further simulation analysis.
- In terms of theoretical measurements, the theoretical calculations of the resonance for the measurement frequencies in the 6 and 7 GHz frequency range was close to the experimental outcome. Again, the slight difference was observed in the case of the resonant peak of S_11_ measurement.
- A non-conventional phenomena of increase in frequency with permittivity was observed and could be attributed to the interfacial polarisation or significant dependence of samples on temperature or frequency parameters as discussed in Sect. 3.4. However, further study is required to explore this in more detail and at this occasion it is not clear which of the above factors are making the samples to respond strangely.
- As mentioned earlier, there is a need to capture all the data and built up a database for the future reference to allow the potential development of the analysis technique up to a prototype level. Pulling out the data and making comparisons between various set of data would also allow to target anomalies in the existing technique and help in optimising the development of the new sensor system for more accurate and predictable results.

Development of microwave sensing technique could bring a significant benefit to the silver manufacturing industry. However, the key is to develop the sensor and sensing technique that can target the industry requirements in a simple manner. Empowering the silver industry with instantaneous analysis technique can reduce the time spend on the analysis through conventional methods, can reduce the labour intensive work, improve the quality of the product and reduce the costs associated with the conventional analysis techniques in a precise manner. The cost effectiveness can be achieved by focusing the analysis around a narrow band of frequency.
